# After-Thoughts

**Published:** 1896-06

**Authors:** 


### Editorial.

### AFTER-THOUGHTS.

It is not unusual for writers of papers to generalize upon insufficient data, or, with positive results, reach conclusions not at all warranted, and, in degree, foreign from the real issue sought to be elucidated by elaborate experiments. This is a fault common to all scientific workers, probably without exception. The nature of this work tends to a certain optimistic view, and, in a measure, clouds the intellectual powers for the time being, then follows a period of rest, and the individual finds that his enthusiasm has carried him beyond legitimate conclusions. Hence, after-thoughts often lead to explanations that detract materially from the original work, as it leads to a feeling in many minds that, inasmuch as the generalization has proved so defective, therefore the facts deduced from elaborate experimentation must be equally faulty. Such a conclusion is, probably, never warranted. Individuals are very differently constituted. The exact investigator, capable of guarding against failure in the minutest detail of his work, may be mentally incapacitated from drawing correct conclusions as to the general effect of the facts secured. It is questionable whether many are so thoroughly mentally rounded that they are equally capable in both these directions. Readers of journals must have had this forced upon their notice, but while it certainly remains a part of human limitation it in no sense discredits good work.

The effect that these crude conclusions have is, however, injurious in that they influence many minds who, attracted by the original brilliant results, will read, but will probably never see, or, if they have seen, will not heed the after-thoughts which may modify the earlier conclusions. In this way erroneous ideas become fastened on our profession, influencing each one of us for good or ill in daily practice.

The illustrations of this are many throughout dental literature, but it is not often that more marked examples are observed than in two very prominent and recent cases, both of which have greatly influenced the thoughts of the active minds in dentistry, possibly more so than in any other direction in many years. We allude to that incisive and, in many respects, the most influential paper recently published upon the etiology of pyorrhœa alveolaris, by Professor C. N. Peirce, and also to that other series of papers by Dr. G. V. Black begun in the *Dental Cosmos*, May, 1895, the most elaborate in experimental details of probably any other in the past decade.

In the paper of Dr. Peirce we have certain results given with a positiveness of assertion born of a thorough conviction that the observations support the author in his hypothesis, and it must be said of the writer of that paper that he has not, so far as we are aware, changed his opinions or modified his conclusions, but this cannot be said of all who have coincided with him. The editor of the *Dental Cosmos* was one of the earliest and warmest supporters of the "gouty origin" of pyorrhœa, and of the results obtained by the chemists aiding Professor Peirce, but he has so recently modified his views (editorial, April, 1896) that some who have never favored that theory have cause to wonder whether the foundation of this hypothesis has not been rudely shaken.

In the original paper by Professor Peirce the following tests were given:

"1. The hydrochloric acid test. 2. The dry or the destructive distillation test. 3. The murexid test." The editor of the *Dental Cosmos*, in the February number, 1894, thus writes of these experiments: "*Careful analysis, both chemical and microscopic*, of concretions from or near the apices of tooth-roots, in typical pyorrhœa cases *demonstrated the uniform presence of uric acid and its salts. This fact alone* is of the *utmost significance*, for by it we not only are confronted with a *fundamental chemical difference in the two classes of concretions*, . . . but we are furnished with a valuable confirmatory evidence of their difference in origin." (Italics ours.) Yet we find, notwithstanding this complete endorsement of Professor Peirce's experiments, that in an article, September, 1894, the editor, in drawing some distinctions between the work of Dr. Black and that of Dr. Peirce in this direction, makes this remark, "It should be noted in this connection that the murexid reaction is purely a qualitative test for detecting the presence of uric acid, and gives but an imperfect basis for its quantitative estimation." In an editorial in the April number, 1896, he uses the following language: "The strong probability is that *the methods of analysis pursued by the experts who did the work for Dr. Talbot were inaccurate. The murexid reaction is easily obtained* where we have to deal with considerable amounts of uric acid or its salts. The case is quite different when minute traces of these compounds are to be detected. The directions usually given in the books for detecting uric acid by the murexid test are, as a rule, *faulty when applied to the material under consideration.*"

While the editor subsequently disclaims any intention of reflecting on the skill of the chemists or the honesty of Dr. Talbot, it seems to our comprehension that he certainly does the former.

If, then, the murexid test is so faulty, what becomes of all that has previously been said in relation to uric acid and its salts in connection with pyorrhœa? It would seem that the work heretofore done must all be repeated, and we think, in justice to Dr. Peirce and those who side with him, that the editor of the *Dental Cosmos* should explain what constitutes a positive test for uric acid, that thereby we may all be benefited. As the case stands at present, it looks very much as though it had resolved itself into discrediting the witnesses on the other side to sustain an untenable hypothesis. We do not care to entertain this view of it, but certainly some explanation is needed, or it may be thought, by careless readers, that the Chicago experts in chemistry are unworthy of credence. This we are not prepared to believe, but fear that the difficulty may be found in the fact that the report of the Chicago chemists has been very unsettling, requiring an effort at explanation. What the profession desires is the truth in the matter, and cares very little for the personal side of the controversy.

A further illustration of our heading may be found in two quotations given from the pen of Dr. G. V. Black, the first from the May (1895) number of the *Dental Cosmos.* We took occasion, at the proper time, to criticise the following assertion as being calculated to mislead and unsettle well-established principles of practice, and we are pleased that Dr. Black felt the necessity of making a subsequent explanation. His after-thought in this case is decidedly his best thought from our point of view.

On page 416 of the aforesaid number will be found this remarkable statement: "*There is no basis for the supposition that some teeth are too soft or too poorly calcified to bear filling with gold or other metal in use for that purpose, since all are found to be abundantly strong.*" (Italics ours.)

His after-thought is expressed in a paper read before the New York Odontological Society, in January last, *Dental Cosmos*, April, 1896, in which the following good advice is given: "*We are not always justified in making a gold filling immediately for a sensitive child* simply because the tooth is sufficiently strong. *The condition of the pulp of the tooth may not justify such a course, or even the dentine may be too sensitive.*"

When properly explained, there is really no conflict between these statements, but the first, left as it originally stood, became a stumbling-block in the way of many an earnest practitioner.

No intelligent person can contend against frequent differences in the mental view of things. The unchangeable person is not a proper part of the close of the nineteenth century, but would seem to be a mental left-over from the fifteenth. This article, therefore, is in no sense a criticism of these changes, on the contrary, it would seek to emphasize the importance of after-thoughts whenever there has been a tendency to overstate results. That which seems to be most needed is that greater care should be taken in our experimental work, so that it shall be nearly free from inaccuracies, and thus avoid, as much as possible, immature conclusions, especially where they conflict with established clinical experience.

